# Post-traumatic stress disorder symptomatology is associated with insomnia among women engaged in opioid use disorder treatment with buprenorphine

**DOI:** 10.1007/s00737-024-01487-5

**Published:** 2024-08-01

**Authors:** Hannah Stadtler, Susie Turkson, Michelle Eglovitch, Dace S. Svikis, Gretchen Neigh, Caitlin E. Martin

**Affiliations:** 1 Department of Anatomy and Neurobiology, Virginia Commonwealth University, Richmond, VA USA; 2 Department of Psychology, Virginia Commonwealth University, Richmond, VA USA; 3 Institute for Drug and Alcohol Studies, School of Medicine, Virginia Commonwealth University, Richmond, VA USA; 4 Department of Obstetrics and Gynecology, School of Medicine, Virginia Commonwealth University, 1101 East Marshall Street, 11th Floor, Room 11-027, Box #980034, 23298 Richmond, VA USA

**Keywords:** PTSD, Trauma, Insomnia, Buprenorphine, Women

## Abstract

This study aimed to explore the association between the degree of PTSD symptomatology and severity of insomnia symptoms in a clinical sample of women receiving buprenorphine for OUD. PTSD symptomatology was assessed via the PCL-5, and insomnia symptoms were determined via the Insomnia Severity Index. Analyses indicated that more participants experiencing clinically significant PTSD symptomatology also reported insomnia symptoms than their counterparts. Future work should investigate how holistic care (e.g., trauma-informed approaches) that addresses the overlap between trauma and sleep disturbance could inform gender-specific OUD treatment strategies in the overdose crisis.

## Introduction

Medications for opioid use disorder (MOUD), like buprenorphine and methadone, reduce risk of overdose and improve quality of life for people with opioid use disorder (OUD) (Larochelle et al. 2018). However, return to non-prescribed opioid use and OUD treatment discontinuation rates remain high. Sleep disturbances, especially insomnia (a chronic condition defined by difficulty falling asleep or staying asleep), are common among individuals in MOUD treatment (Wilkerson and McRae-Clark 2021). MOUD patients face multiple biopsychosocial risk factors for insomnia, ranging from community-level social determinants like unstable housing to individual-level clinical variables (Eglovitch et al. 2023). Considering an example of the latter, MOUD treatments themselves are associated with neurobiological changes in sleep architecture (Huhn and Finan 2022). Thus, exploring the complexity underlying insomnia within the context of MOUD engagement could inform treatment innovations (Eckert and Yaggi 2022).

One major risk factor for sleep disturbances, and particularly for insomnia, is post-traumatic stress disorder (PTSD). PTSD is a psychiatric condition that may occur in people who have experienced or witnessed a traumatic event or set of circumstances. PTSD has been proposed as having a bidirectional relationship with sleep issues: on days when PTSD symptoms are worse, sleep is worse, which further exacerbates PTSD symptoms and perpetuates this cycle (Slavish et al. 2022). However, the relationship between PTSD and insomnia symptoms among people receiving MOUD has yet to be elucidated. This relationship represents a promising area for further investigation.

Further, differences related to sex and gender-related factors exist in PTSD, insomnia, and OUD. The lifetime prevalence of PTSD in women is twice the documented prevalence in men, and women with PTSD are more susceptible to experiencing sleep disturbances compared to men (Kilpatrick et al. 2013). OUD treatment trajectories differ between men and women, such as being differentially impacted by trauma histories. For example, interpersonal trauma has been found to be associated with return to substance use among women, but not among men, in buprenorphine-based OUD treatment (Martin et al. 2022).

Therefore, in a clinical sample of women engaged in OUD treatment with buprenorphine, the present study: (1) reports on the prevalence of patient-reported insomnia symptoms in patients with clinically significant and subthreshold PTSD symptomatology, and (2) examines the association between the severity of PTSD symptomology and severity of insomnia symptoms. While this study was exploratory, we hypothesized that women receiving buprenorphine for OUD with comorbid PTSD symptomatologies would report worse sleep than their counterparts.

## Methods

### Participants and Study Design

This study was a secondary analysis from a parent study assessing insomnia among individuals receiving buprenorphine in outpatient OUD treatment. The parent study was conducted from February 2022-September 2023. Participants within the parent study were assigned female sex at birth, diagnosed with OUD, not pregnant nor within 6 weeks postpartum, aged 18–65, and stabilized on buprenorphine for at least 6 weeks. Detailed methods are described in Eglovitch et al. (2023). All parent study participants identified as cisgender; therefore, we use the term ‘women’ in the present study to refer to cisgender women.

### Measures

The primary outcome of patient-reported insomnia symptoms were determined via the Insomnia Severity Index (ISI). The ISI is a 7-item scaled self-report tool which assesses overall sleep quality. Total scores range from 0 to 28 with higher scores indicating worse insomnia. A cutoff score of *> =* 10 was used to identify clinically significant insomnia (Morin et al. 2011). We also report on the prevalence of individuals endorsing individual ISI items to explore specific domains of sleep disturbance.

Clinically significant PTSD symptomatology was assessed via the PCL-5. The PCL-5 assesses the twenty DSM-5 symptoms of PTSD, with a self-report rating scale of 0–4 for each question. A total symptom severity score is obtained from combining the scores for each of the 20 items, and ranges from 0 to 80, with higher scores indicating greater PTSD symptom severity (Weathers et al., n.d.). A cutoff score of 31 was used to define clinically significant versus subthreshold PTSD symptomatology (Weathers et al., n.d.).

Demographic variables were assessed by self-report on questionnaires. The degree of social support was determined with the question: “In a typical week, how often do you get together with friends or relatives,” with answer choices: rarely/never, sometimes, or usually/always.

### Data Analysis

Analyses were conducted with SPSS (version 28). For the first study objective, descriptive statistics were calculated by PTSD symptomatology groups. For the second objective, a multiple linear regression analysis examined the association between degree of PTSD symptomatology and insomnia symptom severity. In addition to PCL-5 score, four other variables were included in the model with the outcome being ISI total score: age, social support, receipt of behavioral treatment and psychiatric medication, which were included based on existing literature (Slavish et al. 2022).

## Results

### Study sample

Among the total sample of 79 women participants (recruitment rate = 76.0%), 46 reported clinically significant PTSD symptomology (58.2%), and 33 reported subthreshold PTSD symptomatology (41.8%) (Table 1). The mean PCL-5 score among participants with clinically significant PTSD symptomology was 54.6 (SD = 12.3), and the mean score among participants with subthreshold PTSD symptomology was 13.9 (SD = 10.0) (range: 0–80).

PTSD groups were similar across sociodemographic variables. Both PTSD groups were receiving comparable doses of buprenorphine (M = 24 mg for both groups) and had been in OUD treatment for long durations of time (M = 56.4 months and 55.2 months, respectively). In this ‘real-world’ clinical sample, non-opioid substance use was common, largely nicotine (PTSD symptomatology 71.7%; subthreshold-PTSD symptomatology 60.6%), followed by cannabis (30.4% and 54.5%) and alcohol (21.7% and 15.2%).

### Differences in insomnia symptoms between participants with clinically significant and subthreshold PTSD symptomatology

More participants experiencing clinically significant PTSD symptomatology reported insomnia symptoms than their counterparts, including difficulty falling asleep (87% vs. 60.6%), difficulty staying asleep (89.1% vs. 69.7%) and having problems with waking up too early (93.5% vs. 66.7%). Additionally, total ISI scores were higher among participants with clinically significant PTSD symptomatology than those with subthreshold PTSD symptomatology (17.6 vs. 10.1).

**Table 1:** Sociodemographic, clinical, and sleep characteristics of study sample

	Clinically significant PTSD symptomatology (*n* = 46) M (SD) or *N* (%)	Subthreshold PTSD symptomatology (*n* = 33) M (SD) or *N* (%)
**Demographics**		
Age (years)	35.9 (8.9)	38.6 (9.4)
Race White Black Other	26 (56.6%) 17 (37.0%) 3 (6.5%)	22 (66.7%) 8 (24.2%) 3 (9.1%)
Ethnicity Hispanic/Latinx Not Hispanic/Latinx	3 (6.5%) 43 (93.5%)	1 (3.0%) 32 (97.0%)
Employment Employed Unemployed Disabled	12 (26.1%) 25 (54.3%) 9 (19.6%)	11 (33.3%) 16 (48.5%) 6 (18.2%)
Insurance Public Private None	45 (97.8%) 1 (2.2%) 0 (0%)	26 (78.8%) 5 (15.2%) 2 (6.1%)
Education *≤* High school > High school	31 (67.4%) 15 (32.6%)	21 (63.6%) 12 (36.4%)
Social support (% yes)	38 (82.6%)	27 (81.8%)
**Clinical Variables**		
Behavioral treatment (% yes)	27 (58.7%)	11 (33.3%)
Psychiatric Medication	40 (87.0%)	16 (48.5%)
Past 28-day non-prescribed substance use (% yes) Nicotine Cannabis Alcohol Cocaine Heroin/Fentanyl Amphetamines Sedatives	33 (71.7%) 14 (30.4%) 10 (21.7%) 4 (8.7%) 1 (2.2%) 2 (4.3%) 1 (2.2%)	20 (60.6%) 18 (54.5%) 5 (15.2%) 5 (15.2%) 0 (0%) 1 (3.0%) 0 (0%)
**OUD Variables**		
Dose of buprenorphine (mg, range)	24 (4–30)	24 (6–18)
Length of time in treatment (months)	56.4 (46.3)	55.2 (46.5)
**Sleep Variables**		
Clinically significant insomnia symptoms (ISI score > = 10)	40 (87.0%)	16 (48.5%)
Total ISI score (range: 0–28)	17.6 (6.2)	10.1 (7.8)
Difficulty falling asleep (% yes)	40 (87.0%)	20 (60.6%)
Difficulty staying asleep (% yes)	41 (89.1%)	23 (69.7%)
Problems waking up too early (% yes)	43 (93.5%)	22 (66.7%)

### Association between PTSD symptomatology and insomnia symptom severity

The multiple regression model significantly predicted insomnia scores, *F* (5, 64) = 4.12, *p* = .003, *R*^2^ = 0.24. Together, these predictors accounted for 24.4% of the variance in insomnia scores. Degree of PTSD symptomology, *t* (69) = 3.325, *p* = .001 was significantly associated with insomnia symptom severity (Table 2).

**Table 2:** Predictors of insomnia severity

	Insomnia Severity Index (ISI) Total Score				
				95% Confidence Interval for B	
	Beta	t	Sig.		
PCL-5 Total Score	4.09	3.33	0.001	0.062	0.249
Age	0.082	0.734	0.465	− 0.118	0.225
Social support	0.047	0.390	0.698	-2.031	3.017
Behavioral treatment	0.034	0.281	0.780	-3.236	4.294
Psychiatric medication	0.152	1.251	0.215	-1.561	6.794

## Discussion

We found that PTSD was associated with insomnia symptoms in a clinical sample of women in outpatient OUD treatment. Of note, our participants had been receiving buprenorphine on average for over 4 years across PTSD groups, underscoring how mental health and sleep problems can persist across stages of OUD treatment and recovery. These data indicate that targeting PTSD within the context of emerging sleep health interventions could more broadly impact health outcomes among women receiving MOUD treatment. Notably, insomnia-related sleep disturbances were highly prevalent across both groups with clinically significant and subthreshold PTSD symptomologies, consistent with existing literature (Huhn and Finan 2022). Collectively, findings emphasize the importance of considering sleep disturbances in the context of long-term mental health strategies within MOUD treatment settings.

Study findings support the notion that, because of the overlapping influences of PTSD on sleep, and sleep disturbances on behavioral treatments, individuals on MOUD likely benefit from holistic care that aims to address both PTSD and insomnia within the context of OUD treatment. An example is trauma informed care, which is a healthcare approach that frames care around a patient’s unique history of trauma in order to address an individual’s healthcare needs (Center for Substance Abuse Treatment (US), 2014). Trauma informed care includes implementing trauma screening, creating an inclusive environment to promote patient disclosure, and improving sensitive provider-patient relationships (Center for Substance Abuse Treatment (US), 2014). This is especially important for women in MOUD care, due to their particularly high burden of PTSD and insomnia (Slavish et al. 2022). Our study suggests that emerging gender-specific therapeutics targeting insomnia among MOUD patients should prioritize trauma informed care in their development and investigations to ensure patient-centeredness in clinical applicability and research.

There were several limitations within this analysis. This study relies on cross-sectional self-report data. Additionally, many participants endorsed ongoing substance use, reflective of our ‘real world’ sample of individuals in MOUD treatment. Furthermore, women were maintained on buprenorphine longer than the average length of retention in MOUD treatment, which is typically less than 6 months (Wakeman et al. 2020), limiting generalizability. However, our specialized study sample provides unique insight into a population highly engaged in care yet still in need of tailored interventions to improve treatment outcomes. Future research could assess if sleep disturbances change as PTSD symptomatology changes in women on MOUD, which would give further insight into the relationship among multiple risk factors for sleep disturbances.

## Conclusions

This study investigated associations between PTSD symptoms and insomnia in a clinical sample of women engaged in outpatient OUD treatment with buprenorphine. We found that clinically significant PTSD symptomatology in this group is associated with severity of insomnia symptoms. Findings highlight the need to investigate best practices to integrate person-centered, gender-informed, targeted treatments for co-morbid conditions, such as insomnia, among this clinical population.

## Data Availability

Research data are not shared.
